# Author Correction: Attosecond coherent control of free-electron wave functions using semi-infinite light fields

**DOI:** 10.1038/s41467-019-08996-3

**Published:** 2019-03-01

**Authors:** G. M. Vanacore, I. Madan, G. Berruto, K. Wang, E. Pomarico, R. J. Lamb, D. McGrouther, I. Kaminer, B. Barwick, F. Javier García de Abajo, F. Carbone

**Affiliations:** 1 Institute of Physics, Laboratory for Ultrafast Microscopy and Electron Scattering (LUMES), École Polytechnique Fédérale de Lausanne, Station 6, 1015 Lausanne, Switzerland; 2 Department of Electrical Engineering, Technion—Israel Institute of Technology, Haifa, 3200003 Israel; 3 SUPA, School of Physics and Astronomy, University of Glasgow, Glasgow, G12 8QQ UK; 4 Ripon College, 300 W. Seward St., Ripon, WI 54971 USA; 5 ICFO-Institut de Ciencies Fotoniques, The Barcelona Institute of Science and Technology, 08860 Castelldefels, Barcelona Spain; 6 ICREA-Institució Catalana de Recerca i Estudis Avançats, Passeig Lluís Companys 23, 08010 Barcelona, Spain

Correction to: *Nature Communications*; 10.1038/s41467-018-05021-x; published online 12 July 2018

The authors became aware of a mistake in the original version of this Article. Specifically, an extra factor γ was incorrectly included in a number of mathematical equations and expressions. As a result of this, the following changes have been made to the originally published version of this Article:

Equation 1 originally incorrectly read:

$$\beta = \left( {e\gamma {\mathrm{/}}\hbar \omega } \right){\int} {dz\,{\cal E}_z(z)e^{ - i\omega z/v}} .$$

The correct form of Equation 1 is:

$$\beta = \left( {e{\mathrm{/}}\hbar \omega } \right){\int} {dz\,{\cal E}_z(z)e^{ - i\omega z/v}} .$$

Equation 3 originally incorrectly read:

$$\beta \approx \left( {{\mathrm{i}}e\gamma /\hbar \omega } \right)\left[ {\frac{{{\cal E}_z^{\mathrm{i}}}}{{\omega /v - k_z^{\mathrm{i}}}} + \frac{{{\cal E}_z^{\mathrm{r}}}}{{\omega /v + k_z^{\mathrm{r}}}}} \right].$$

The correct form of Equation 3 is:

$$\beta \approx \left( {{\mathrm{i}}e{\mathrm{/}}\hbar \omega } \right)\left[ {\frac{{{\cal E}_z^{\mathrm{i}}}}{{\omega {\mathrm{/}}v - k_z^{\mathrm{i}}}} + \frac{{{\cal E}_z^{\mathrm{r}}}}{{\omega /v + k_z^{\mathrm{r}}}}} \right].$$

The seventh sentence of the ‘Theory of ultrafast electron–light interaction’ section of the Methods originally incorrectly read ‘Putting these elements together, the Schrödinger equation reduces to $\left( {{\mathbf{v}}.\nabla + \partial /\partial t} \right)\phi = \frac{{ - {\mathrm{i}}e\gamma {\mathbf{v}}}}{{\hbar c}}.{\mathbf{A}}\,\phi$, where $\gamma = 1/\sqrt {1 - \nu ^2/c^2}$, which admits the rigorous solution $\phi \left( {{\mathbf{r}},t} \right) = \phi _0\left( {{\mathbf{r}} - {\mathbf{v}}t} \right){\mathrm{exp}}\left[ {\frac{{ - {\mathrm{i}}e\gamma {\mathbf{v}}}}{{\hbar c}}.\mathop {\int }\nolimits_{ - \infty }^t dt^\prime {\mathbf{A}}\left( {{\mathbf{r}} + {\mathbf{v}}t^\prime - {\mathbf{v}}t,\,t\prime } \right)} \right]$.’ The corrected version states instead ‘Putting these elements together, the Schrödinger equation reduces to $\left( {{\mathbf{v}}.\nabla + \partial /\partial t} \right)\phi = \frac{{ - {\mathrm{i}}e{\mathbf{v}}}}{{\hbar c}}.{\mathbf{A}}\,\phi$, which admits the rigorous solution $\phi \left( {{\mathbf{r}},t} \right) = \phi _0\left( {{\mathbf{r}} - {\mathbf{v}}t} \right){\mathrm{exp}}\left[ {\frac{{ - {\mathrm{i}}e{\mathbf{v}}}}{{\hbar c}}.\mathop {\int }\nolimits_{ - \infty }^t dt^\prime {\mathbf{A}}\left( {{\mathbf{r}} + {\mathbf{v}}t^\prime - {\mathbf{v}}t,\,t\prime } \right)} \right]$.’

The tenth sentence of the same paragraph originally read ‘Inserting this expression into Eq. (4), we find the solution $\phi \left( {{\mathbf{r}},t} \right) = \phi _0\left( {{\mathbf{r}} - {\mathbf{v}}t} \right)e^{ - {\cal B} + {\cal B} \ast }$, where ${\cal B}\left( {{\mathbf{r}},t} \right) = \frac{{e\gamma {\mathbf{v}}}}{{\hbar \omega }}.\mathop {\int }\nolimits_{ - \infty }^t dt^\prime \,\vec {\cal E}_0\left( {{\mathbf{r}} + {\mathbf{v}}t^\prime - {\mathbf{v}}t,\,t^\prime } \right){\mathrm{e}}^{ - {\mathrm{i\omega }}t^\prime }$.’ The corrected version states instead states that ‘${\cal B}\left( {{\mathbf{r}},t} \right) = \frac{{e{\mathbf{v}}}}{{\hbar \omega }}.\mathop {\int }\nolimits_{ - \infty }^t dt^\prime \,\vec {\cal E}_0\left( {{\mathbf{r}} + {\mathbf{v}}t^\prime - {\mathbf{v}}t,\,t^\prime } \right){\mathrm{e}}^{ - {\mathrm{i\omega }}t^\prime }$’

Equation 5 originally incorrectly read:

$$\beta \left( {\mathbf{r}} \right) = \frac{{e\gamma }}{{\hbar \omega }}\mathop {\int }\nolimits_{ - \infty }^z dz^\prime \,{\cal E}_{0z}\left( {x,\,y,\,z^\prime } \right){\mathrm{e}}^{ - {\mathrm{i\omega }}z^\prime /v}.$$

The correct form of Equation 5 is:

$$\beta \left( {\mathbf{r}} \right) = \frac{e}{{\hbar \omega }}\mathop {\int }\nolimits_{ - \infty }^z dz^\prime \,{\cal E}_{0z}\left( {x,\,y,\,z^\prime } \right){\mathrm{e}}^{ - {\mathrm{i\omega }}z^\prime {\mathrm{/}}v}.$$

Finally, in the Supplementary Information, supplementary Equation 1 originally incorrectly read:

$$\beta \approx \frac{{iev\gamma {\cal E}_0}}{{\hbar \omega ^2}}\left[ {\frac{{sin\delta }}{{1 - (v/c)cos\delta }} + \frac{{{\mathrm{sin}}(\delta - 2\alpha )}}{{1 + (v/c)cos(\delta - 2\alpha )}}} \right].$$

The correct form of supplementary Equation 1 is:

$$\beta \approx \frac{{iev{\cal E}_0}}{{\hbar \omega ^2}}\left[ {\frac{{sin\delta }}{{1 - (v{\mathrm{/}}c)cos\delta }} + \frac{{{\mathrm{sin}}(\delta - 2\alpha )}}{{1 + (v{\mathrm{/}}c)cos(\delta - 2\alpha )}}} \right].$$

This has been corrected in both the PDF and the HTML versions of the Article. The error does not affect the results or conclusions of the paper. All figures with theoretical data are normalised and are thus unaltered by this factor of γ ≈ 1.39. Therefore the figures did not require correcting.

