# Supplementary material for: Attosecond coherent control of free-electron wave functions using semi-infinite light fields
Source: Nat Commun. 2018 Jul 12;9:2694. doi: 10.1038/s41467-018-05021-x (PMC6043599; doi:10.1038/s41467-018-05021-x)
Supplement: Supplementary file 1 — Supplementary Information [file 41467_2018_5021_MOESM1_ESM.pdf]

Attosecond coherent control of free-electron wave functions using semi-infinite light fields

– SUPPLEMENTARY INFORMATION –

G. M. Vanacore,[1,*] I. Madan,[1,*] G. Berruto,[1] K. Wang,[1,2] E. Pomarico,[1] R. J. Lamb,[3] D. McGrouther,[3] I. Kaminer,[1,2] B. Barwick,[4] F. Javier García de Abajo,[5,6,†] and F. Carbone[1,‡]

[1]*Institute of Physics, Laboratory for Ultrafast Microscopy and Electron Scattering (LUMES), École Polytechnique Fédérale de Lausanne, Station 6, CH-1015 Lausanne, Switzerland*
[2]*Department of Electrical Engineering, Technion - Israel Institute of Technology, Haifa, Israel*
[3]*SUPA, School of Physics and Astronomy, University of Glasgow, Glasgow G12 8QQ, UK*
[4]*Ripon College, 300 W. Seward St., Ripon, WI 54971, United States*
[5]*ICFO-Institut de Ciencies Fotoniques, The Barcelona Institute of Science and Technology, 08860 Castelldefels (Barcelona), Spain*
[6]*ICREA-Institució Catalana de Recerca i Estudis Avançats, Passeig Lluís Companys 23, 08010 Barcelona, Spain*
(Dated: February 22, 2019)

Supplementary Note 1: Momentum conservation for a semi-infinite light field

It is instructive to visualize the space and momentum distribution of light in the presence of a mirror. Supplementary Figure 1a,b shows the instantaneous light electric field as a function of distance along the electron propagation direction. Without the mirror, the space Fourier transform (Supplementary Figure 1c) consists of two Dirac δ-functions peaked at wavenumbers $k = \pm k_{\rm L} = \pm\omega/c$, where ω is the light frequency and c is speed of light in free space. Coupling of light to a free electron is then forbidden in vacuum because the absorption or emission of a photon involves a momentum exchange[1] $\hbar\Delta k_{\rm e} \sim \pm\hbar\omega/v$ (here, $v = 0.695\,c$ for 200 keV electrons) that does not overlap with the photon momentum. However, in the presence of a mirror the interruption of light propagation (Supplementary Figure 1b) introduces a finite width in the δ-functions, so the corresponding momentum distribution (Supplementary Figure 1d) acquires a $\sim 1/(k \pm k_{\rm L})$ profile that has a finite overlap with $k = \pm\Delta k_{\rm e}$, therefore enabling light-electron coupling.

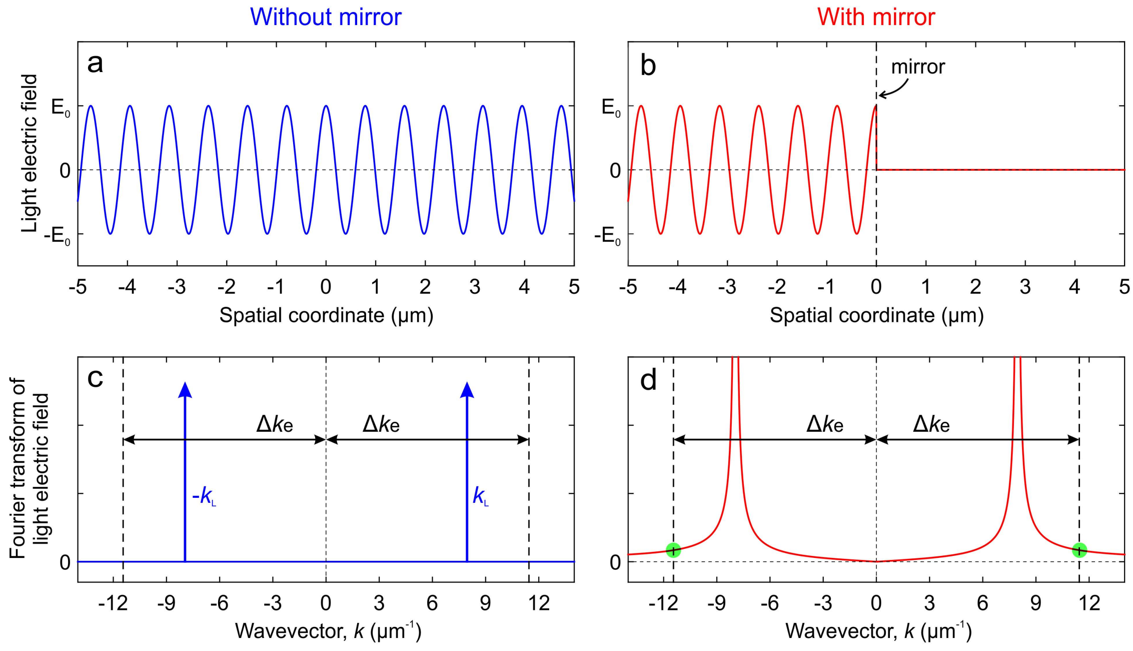

Supplementary Figure 1: Momentum conservation for a semi-infinite vacuum field. (a)-(b) Instantaneous light electric field as a function of distance along the electron propagation direction in the absence (panel a) or presence (panel b) of a mirror. (c)-(d) Space Fourier transforms of the light electric field reported in panels a and b.

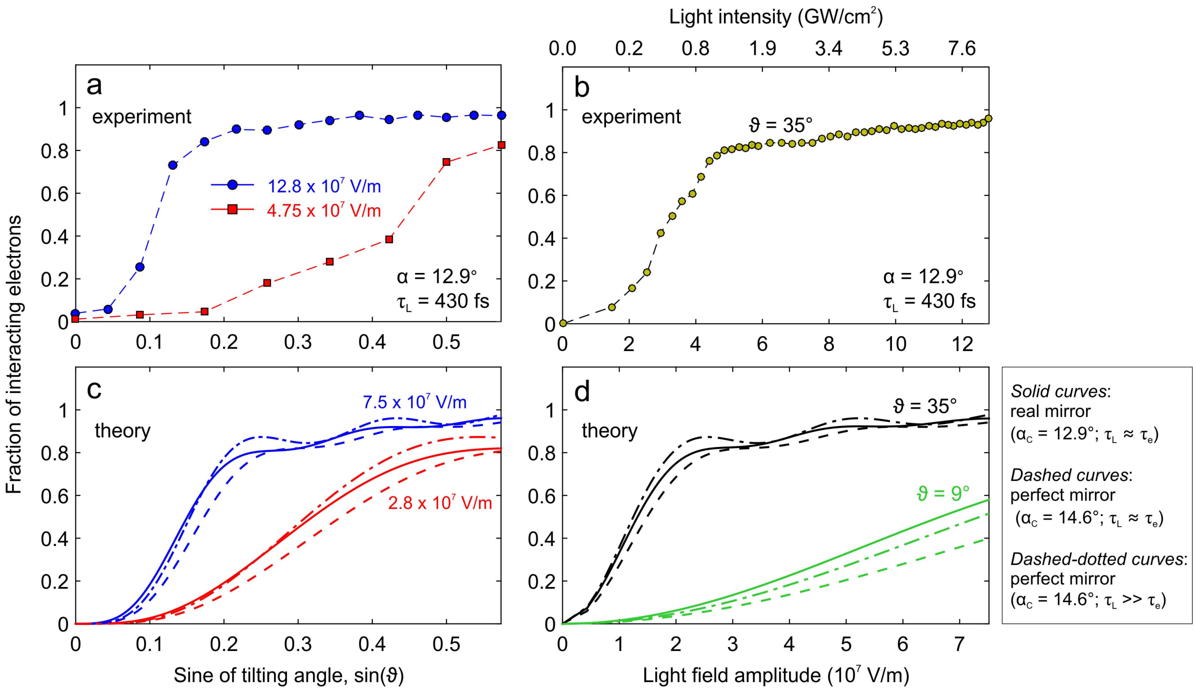

Supplementary Figure 2: Quantification of coupling fraction: experiment and theory. (a)-(b) Experimentally measured fraction of electrons that have gained and lost energy relative to the total number of electrons in the beam when, as a function of tilt angle ϑ (panel a) and light field amplitude (panel b). (c)-(d) Theoretical calculations of the coupling fraction obtained for the same parameters as used in the experiments when changing the tilt angle ϑ (panel c) and the light field amplitude (panel d).

Supplementary Note 2: Quantification of the coupling fraction

A direct quantitative measure of light-electron coupling is provided by the fraction of electrons that have gained and lost energy relative to the total number of electrons in the beam (that is, the *coupling fraction*). This quantity can be readily extracted upon integration of the measured or calculated EELS spectra in Fig. 2 of the main text. Experimental results are represented in Supplementary Figures 2a and 2b as a function of tilt angle ϑ and light field amplitude, respectively. The specific behavior of the coupling fraction can be externally controlled by varying the incident light intensity and/or the tilt angle ϑ, although in both cases the fraction of interacting electrons exhibits a step-like behavior with two regimes: a steep increase is followed by a slower change toward saturation. The physical origin of this behavior is well captured by the theoretical description provided in the Methods section (see Supplementary Figure 2c,d).

We note that the best agreement between theory and experiment is obtained when the light field amplitude is assumed to be around 1.7 times smaller than the value estimated in the experiment. We attribute this factor to unaccounted losses of the laser beam along its path within the microscope, and/or to an experimental underestimate of the laser beam diameter at the mirror. In all figures throughout this paper, we present the amplitudes in accordance with the experimental estimate (that is, when referring to theory, calculations are performed for an amplitude that is 1.7 times smaller than the one shown in the scales).

Supplementary Note 3: Perfect versus real mirror

With a reflectivity $> 99\%$, a metal skin depth $\sim 11\,$nm, smaller than the silver layer thickness $\sim 43\,$nm, and a dielectric function $\approx -30 + 0.4\text{i}$ at the photon energy $\hbar\omega \approx 1.57\,$eV, the mirror performance should be close to that of a perfect mirror. In this approximation, the β parameter (that is, the weighted integral of the light electric field along the electron trajectory, see Methods) for p-polarized light and $\vartheta = 0$ is given by Eq (3) of the main text with $\mathcal{E}_z^{\rm i} = \mathcal{E}_0 \sin\delta$, $\mathcal{E}_z^{\rm r} = \mathcal{E}_0 \sin(\delta - 2\alpha)$, $k_z^{\rm i} = (\omega/c)\cos\delta$, and $k_z^{\rm r} = (\omega/c)\cos(\delta - 2\alpha)$ (see Fig. 1a for the definition of different angles), where $\mathcal{E}_0$ is the incident field amplitude. This leads to

$$\beta \approx \frac{\text{i}ev\mathcal{E}_0}{\hbar\omega^2}\left[\frac{\sin\delta}{1-(v/c)\cos\delta} + \frac{\sin(\delta-2\alpha)}{1+(v/c)\cos(\delta-2\alpha)}\right]. \tag{1}$$

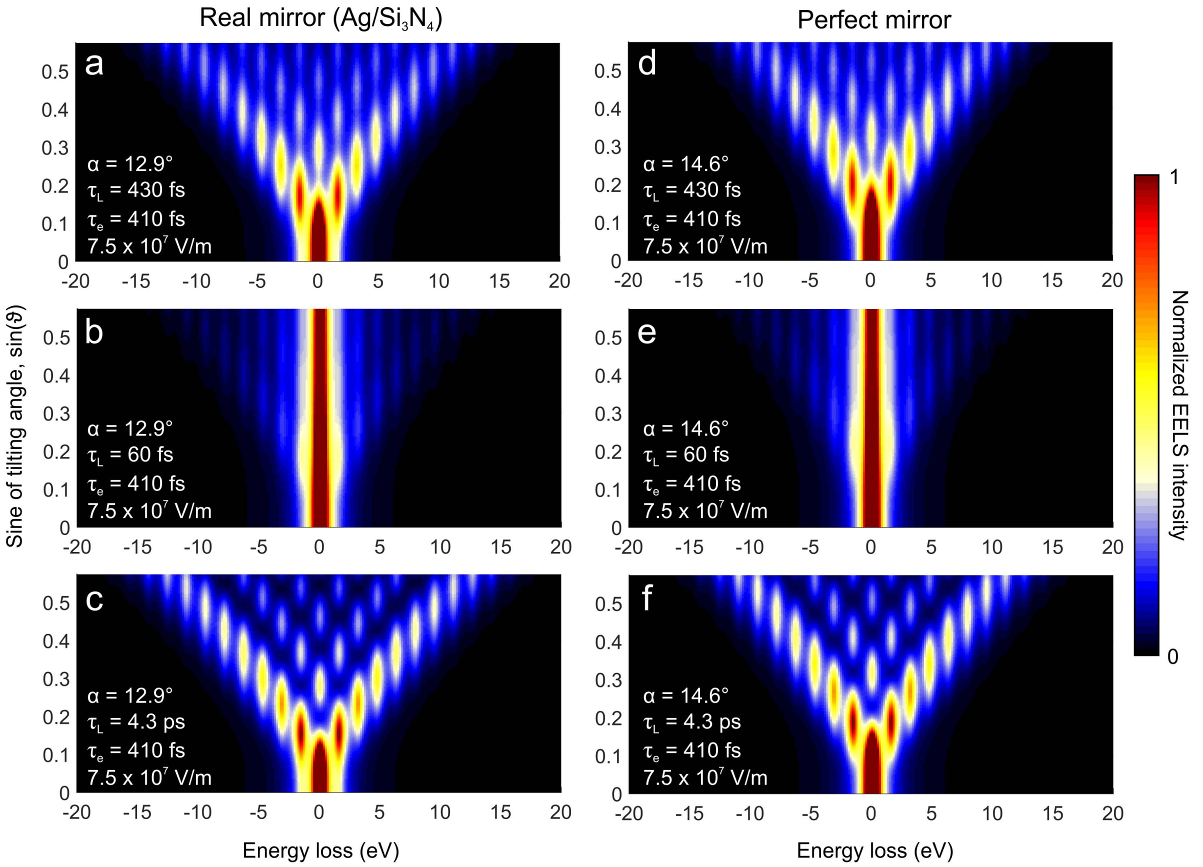

Supplementary Figure 3: Calculations of EELS spectra as a function of the tilt angle ϑ for a perfect metallic mirror (panels a, b and c) and for real materials (panels d, e, and f) in different regimes of temporal width for the electron (τ_e) and light (τ_L) pulses: $\tau_L \approx \tau_e$ (panels a and d), $\tau_L < \tau_e$ (panels b and e), $\tau_L \gg \tau_e$ (panels c and f).

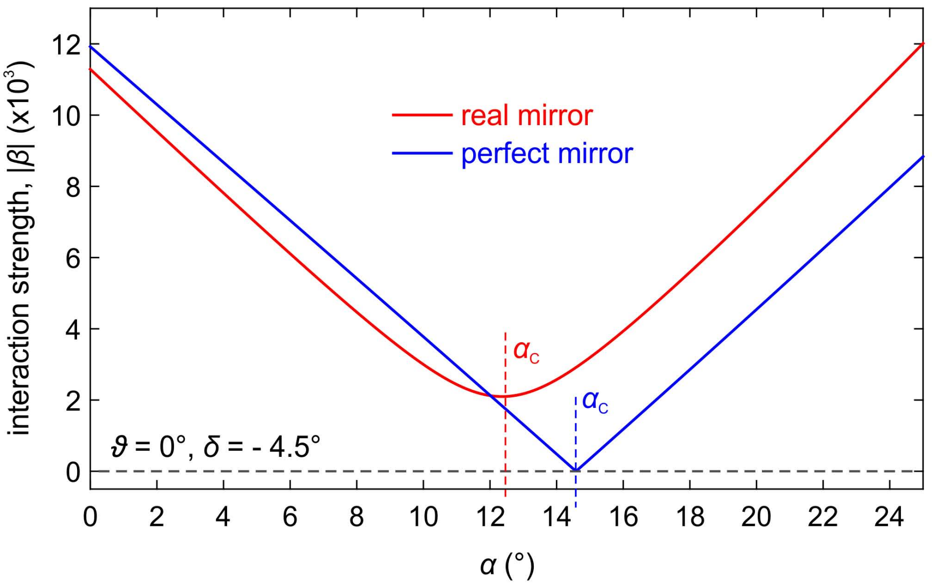

Supplementary Figure 4: Calculated interaction strength $|\beta|$ as a function of tilt angle α for a perfect metallic mirror (blue curve) and for a mirror made of real materials (red curve).

A more involved expression is given for $\vartheta \neq 0$. If we further neglect the finite temporal extension of the light pulses, the probability of exchanging a net number of photons ℓ is given by $J_\ell^2(2|\beta|)$. The theoretical results

based upon these approximations (dashed curves and dashed-dotted curves in Supplementary Figure 2c,d, and color maps in Supplementary Figure 3d-f) are in good agreement with full calculations incorporating real dielectric data for the mirror materials and the finite duration of the light pulses (solid curves in Supplementary Figure 2c,d, and color maps in Supplementary Figure 3a-c).

These results suggest that we can use Supplementary Eq. (1) to obtain a good estimate for the no-interaction tilt angle (that is, $\vartheta = 0$ and $\alpha = \alpha_C$ for which $\beta = 0$). We find

$$\alpha_C = \tan^{-1}\left(\frac{\sin\delta}{\cos\delta - v/c}\right).$$

which establishes a direct relation between the critical angle α_C and δ in the perfect-mirror.

A graphical example of this relation is shown in Supplementary Figure 4, where we plot the dependence of the parameter β on the tilt angle α for perfect and real-materials mirror. In the latter, $\beta = 0$ is never reached and only a global minimum is obtained for a critical angle α_C slightly different from the one obtained for the perfect mirror. Nevertheless, Supplementary Eq. (1) provides a reasonably good description of electron-light interaction, while finer details require using actual dielectric data for the mirror materials.

Supplementary Note 4: Polarization dependence

We have so far discussed results for p-polarized light, which produces the largest light-coupling strength in our setup configuration. For practical applications one can also tune the polarization of the incident light, which provides a high-precision control without changing the sample position. In Supplementary Figure 5 we present an analysis of the coupling fraction as a function of tilt and polarization angles, ϑ and ϕ, respectively. When $\alpha = \alpha_C$ (Supplementary Figure 5a), the electron-photon interaction is negligible at low ϑ for all polarizations and then gradually increases with ϑ until reaching saturation. As anticipated above, coupling is observed to be weakest for s-polarization ($\phi = 0$, incident field parallel to y axis), corresponding to a minimum field component along the electron-beam direction, and reaches maximum values for p polarization ($\phi = \pm 90°$). A more involved dependence on the polarization and tilt angles is observed with $\alpha \neq \alpha_C$ (for example, $\alpha = 0$ in Supplementary Figure 5b), which is still well understood by comparison with the simple theory of Eqs. (1) and (3) in the main text.

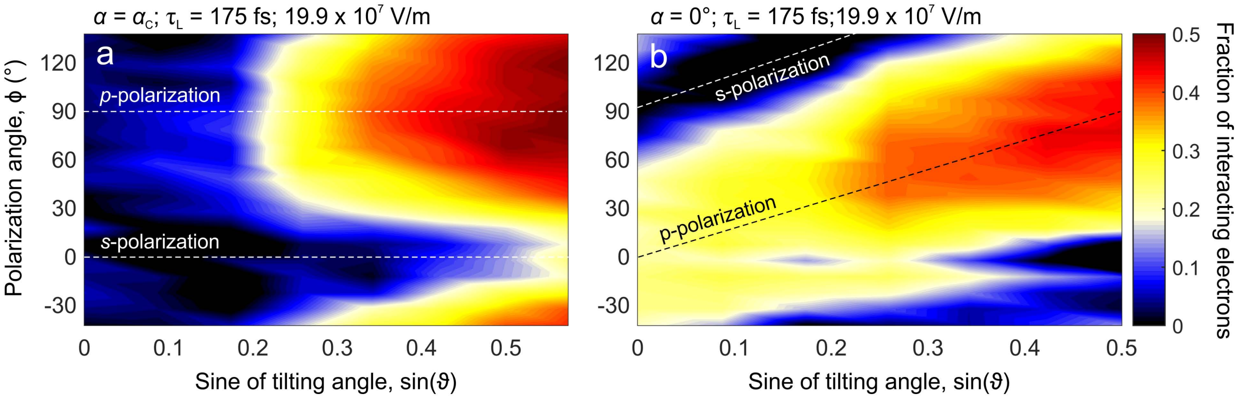

Supplementary Figure 5: Polarization dependence of the coupling fraction. Experimentally measured coupling fraction as a function of tilt and polarization angles, ϑ and ϕ, respectively, for two different condition of the angle α: $\alpha = \alpha_C$ (panel a) and $\alpha = 0°$ (panel b).

Supplementary Note 5: Electron-light interaction with short light pulses

In Supplementary Figure 6 we study light-electron interaction when the light pulse duration is shorter than the electron pulse ($\tau_L = 175$ fs and $\tau_e = 395$ fs). Under these conditions, a substantial portion of the electron beam does not interact with the light, and therefore the probability for multiphoton exchanges with high ℓ is substantially smaller than for low ℓ. Nevertheless, the dependence of the EELS intensity on both the tilt angle ϑ (Supplementary Figure 6a-b) and the light field amplitude (Supplementary Figure 6c) still exhibits a redistribution of the electron probability from the zero-loss peak (ZLP) toward lateral sidebands at multiples of the incident photon energy.

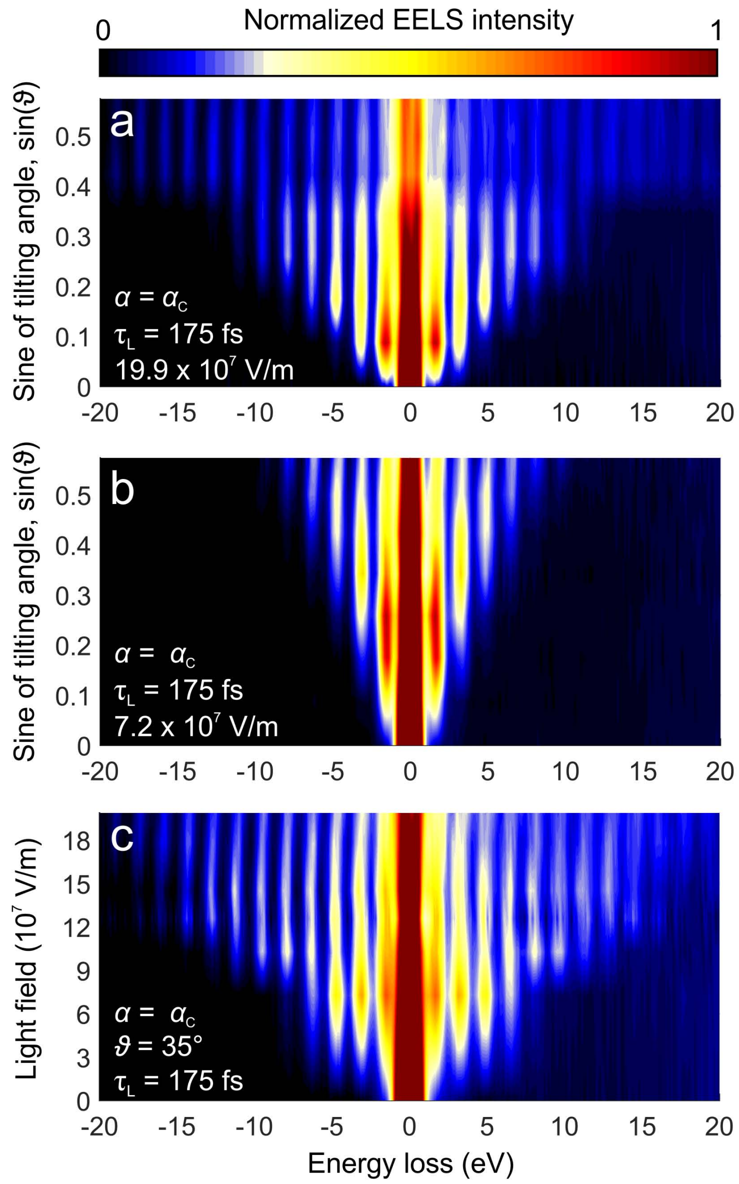

Supplementary Figure 6: Electron-light interaction with short light pulses. Experimentally measured EELS spectra as a function of the tilt angle ϑ (panels a and b) and light field amplitude (panel c) for a light pulse duration τ_{L} shorter than the electron pulse duration τ_{e}.

Supplementary Note 6: Analytical determination of the transversal momentum exchange

In this section we present an analytical derivation of the transferred transversal momentum between the light and the electron. Specifically, we are interested in the transferred momentum components along the directions x and y (see Fig. 1a in the main text), as well as their dependence on sample orientation and light incidence angle. We proceed by expressing the incident and reflected light wave vector in different reference frames after rotation first by an angle ϑ and then by α. We denote (x, y, z) the coordinates in the unrotated frame; (x', y', z') in the frame after rotating by a tilt angle ϑ; and (x'', y'', z'') in the frame after a subsequent rotation by an angle α. These two rotations are described by the relations

$$\begin{aligned} x' &= x\cos\vartheta + z\sin\vartheta, \\ y' &= y, \\ z' &= -x\sin\vartheta + z\cos\vartheta, \end{aligned} \tag{2}$$

and

$$\begin{aligned} x'' &= x', \\ y'' &= y' \cos\alpha - z' \sin\alpha, \\ z'' &= y' \sin\alpha + z' \cos\alpha, \end{aligned} \tag{3}$$

respectively. Combining Supplementary Eqs. (2) and (3), we find

$$\begin{aligned} x'' &= x \cos\vartheta + z \sin\vartheta, \\ y'' &= y \cos\alpha - (z \cos\vartheta - x \sin\vartheta) \sin\alpha, \\ z'' &= y \sin\alpha + (z \cos\vartheta - x \sin\vartheta) \cos\alpha, \end{aligned} \tag{4}$$

from which we directly extract the transformed coordinates of the unit vectors along the unrotated x, y, and z axes as

$$\begin{aligned} (x'', y'', z'')_{\rm x} &= (\cos\vartheta,\ \sin\vartheta \sin\alpha,\ -\sin\vartheta\cos\alpha), \\ (x'', y'', z'')_{\rm y} &= (0,\ \cos\alpha,\ \sin\alpha), \\ (x'', y'', z'')_{\rm z} &= (\sin\vartheta,\ -\cos\vartheta \sin\alpha,\ \cos\vartheta\cos\alpha). \end{aligned} \tag{5}$$

Likewise, we consider the incident light wave vector $(x, y, z)_{\rm L,i} = (0,\ \sin\delta,\ \cos\delta)$ and its transformation using Supplementary Eq. (4):

$$(x'', y'', z'')_{\rm L,i} = (\cos\delta\sin\vartheta,\ \sin\delta\cos\alpha - \cos\delta\cos\vartheta\sin\alpha,\ \sin\delta\sin\alpha + \cos\delta\cos\vartheta\cos\alpha). \tag{6}$$

Now, the film-surface-parallel momentum components (along x'' and y'') must be transferred to the electron during photon absorption because they cannot couple to the film (that is, they are conserved upon light reflection). This gives a wave-vector-transfer contribution

$$(x'', y'', z'')_{\rm L\|} = (\cos\delta\sin\vartheta,\ \sin\delta\cos\alpha - \cos\delta\cos\vartheta\sin\alpha,\ 0),$$

which has coordinates

$$\begin{aligned} (x, y, z)_{\rm L\|} &= \big[(x'', y'', z'')_{\rm L\|} \cdot (x'', y'', z'')_x,\ \ (x'', y'', z'')_{\rm L\|} \cdot (x'', y'', z'')_y,\ \ (x'', y'', z'')_{\rm L\|} \cdot (x'', y'', z'')_z\big] \\ &= \big[\sin\vartheta\cos\alpha(\cos\delta\cos\vartheta\cos\alpha + \sin\delta\sin\alpha), \\ &\quad \sin\delta\cos^2\alpha - \cos\delta\cos\vartheta\sin\alpha\cos\alpha, \\ &\quad \cos\delta(\sin^2\vartheta + \cos^2\vartheta\sin^2\alpha) - \sin\delta\cos\vartheta\cos\alpha\sin\alpha\big] \end{aligned}$$

in the unrotated frame. The corresponding momentum transfer is $k_{\rm L}(x, y, z)_{\rm L\|}$, where $k_{\rm L} = \omega/c$ is the light wavenumber. From the results of Fig. 3 of the main paper, both δ and $\alpha = \alpha_{\rm C}$ are small, so the transfer along y is small compared with that along x, which can be approximated as $q_{\rm T,x} \approx \sin\vartheta\cos\vartheta\cos^2\alpha_{\rm C}$.

Supplementary Note 7: Coherent versus Incoherent electron-light interaction

In Fig. 4a-c of the main text we report a strong temporal modulation of the spectral sidebands in the energy spectrum as a result of electron interaction with a sequence of two mutually-phase-locked light pulses. In order to verify that this effect cannot be assimilated to a simple intensity variation of the exciting light, and neither to an incoherent interaction of the electron beam with the two pulses, we present here several control experiments, additional calculations, and further considerations on our observations.

In our two-pulse scheme we have adopted two phase-locked photon pulses with temporal duration of 60 fs (FWHM) and delayed them more than their temporal cross-correlation in order to minimize their optical interference. For delays between the two pulses ($\Delta_2 - \Delta_1$) above 100 fs, the optical interference induces a total light intensity modulation around $\pm 5\%$ (see Supplementary Figure 7d showing the measured optical cross-correlation between the two optical pulses as a function of their delay time). In what follows we demonstrate that if the interaction between the electron pulse and the light field distribution given by the two optical pulses is incoherent then the modulation of the spectrum, and especially the modulation of the high-energy sidebands, would be only determined by the 5% optical interference and quantitatively in a similar range, in contrast to the experimental observations presented in Fig. 4a-c.

For a proper quantification of the incoherent interaction, we need to consider the temporal profile of the total intensity distribution. This is shown in Supplementary Figure 7a for $\Delta_2 - \Delta_1 = 109$ fs (red curve) and 110.5 fs (blue curve), using the same delay times as reported in Fig. 4 of the main manuscript. In Supplementary

Figure. 7b we simulate the spectral contribution produced by different light intensities, and show that high-energy sidebands become important only at high values of the light intensity (see also Supplementary Figure. 7c, which describes the $\ell = 14$ sideband). This means that the region in between the two pulses, which is mostly modulated by optical interference, would only affect the low-energy sidebands of the spectrum, whereas the high-energy bands would be only slightly modulated by the very small intensity change at the peak of the pulses (see Supplementary Figure 7c). This is in contrast to our experimental observation (shown in Fig. 4a-c and 5a-b of the main manuscript), where a large change is visible especially for the high-energy sidebands. For the $\ell = 14$ peak, the optical interference would result in an intensity modulation of about ±7% (see Supplementary Figure 7e), which is much smaller than the intensity modulation of ±250% observed experimentally (see Fig. 4c and Supplementary Figure 7f). The observed modulation of the energy spectrum as a function of the delay between the two pulses cannot be therefore assimilated to a simple intensity variation of the impinging light as a result of optical interference.

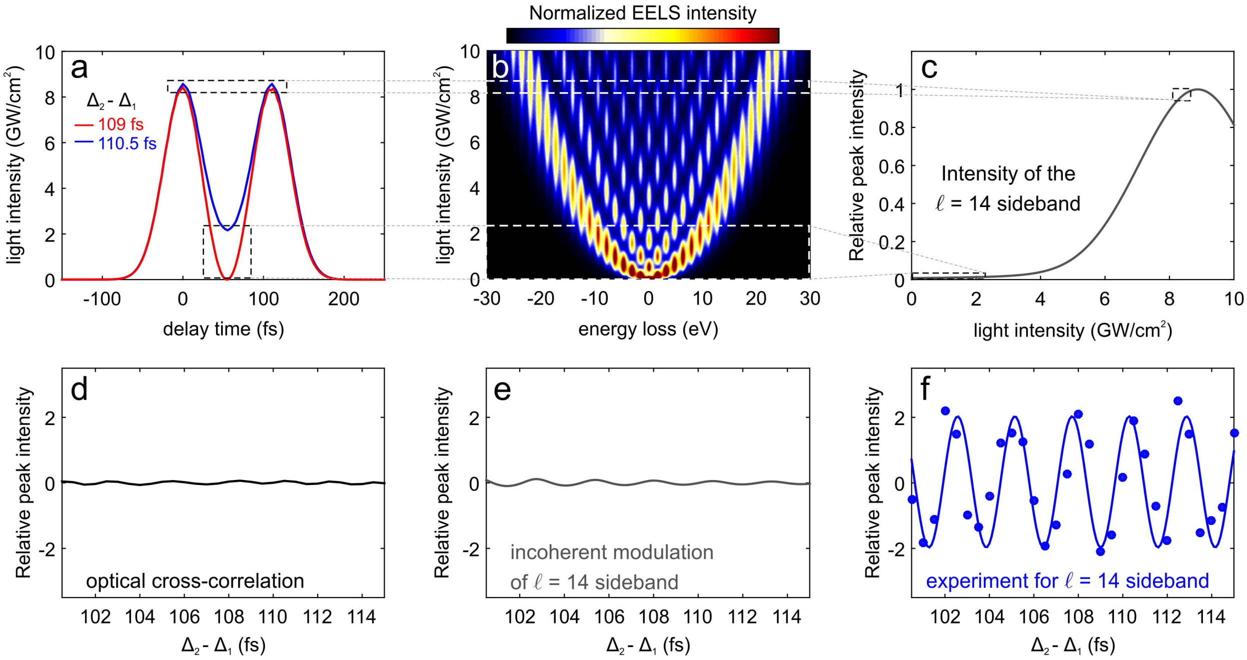

Supplementary Figure 7: (a) Temporal profile of the total intensity light distribution for a delay time between them $\Delta_2 - \Delta_1 = 109\,\text{fs}$ (red curve) and $110.5\,\text{fs}$ (blue curve). (b) Calculated spectral contribution produced by different light intensities. (c) Relative peak intensity of the $\ell = 14$ sideband as a function of light intensity. (d) Measured optical cross-correlation between the two 60-fs photon pulses as a function of $\Delta_2 - \Delta_1$. (e) Calculated temporal modulation of the $\ell = 14$ sideband for incoherent electron-light interaction. (f) Experimentally measured temporal modulation of the $\ell = 14$ spectral sideband.

To further verify this claim, we have performed specific control experiments. In particular, we have monitored the energy spectrum obtained using a single 60-fs-long pulse for a similar light intensity range as used in the two-pulse experiment (see Supplementary Figure 8). Because of the short light pulse duration, at these intensities we are already in a saturation regime and only minor changes are observed when varying the intensity by about 20%. Similarly, we compare the spectra measured with a single light pulse and with two light pulses (see Supplementary Figure 9). For two-pulse illumination we observe the appearance of additional sidebands at the high-energy side (from $\ell = 12$ to $\ell = 17$) with respect to single-pulse excitation, which can only be explained by a coherent modulation imposed by two pulses on the electron wave function. These results further confirm that the modulation of the electron beam discussed in the main manuscript can only be associated with a coherent interaction with the optical field configuration that is properly synthesized using the two mutually-phase-locked light pulses.

As a result of our experimental observations and further control experiments, we can conclude that in our experiments the intrinsic temporal coherence of the single-electron wave function should amount to several tens of femtoseconds. When discussing electron coherence we need to bring about an important consideration: the value of the FWHM of the zero loss peak (ZLP), which is typically described by a Gaussian (inhomogeneous) broadening of 0.9-1 eV, cannot be correlated to the longitudinal electron coherence, which is instead determined by the homogeneous (Lorentzian) energy spread of each single-electron wave function. Under realistic experimental conditions, electrons can be photoemitted with slightly different kinetic energies, as determined by the difference between the photon energy and the work function of the source modulated by the joint density of

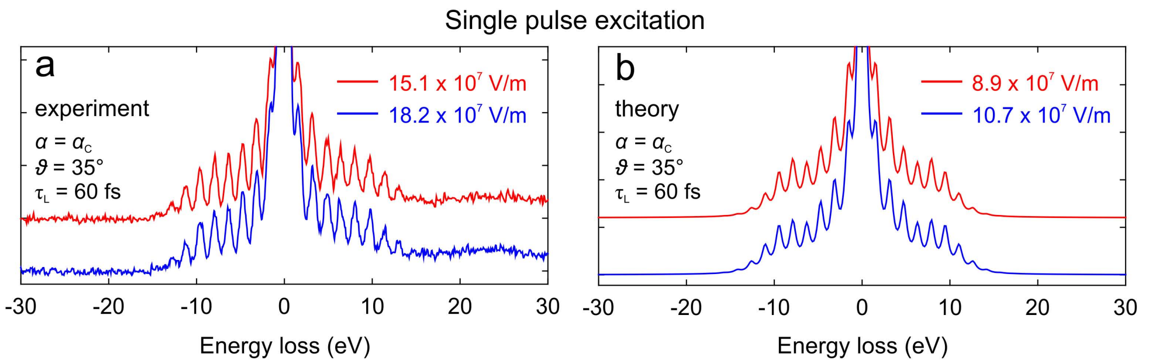

Supplementary Figure 8: Measured (panel a) and calculated (panel b) single-pulse EEL spectra at high light intensity and short pulse duration when varying the intensity by about 20%.

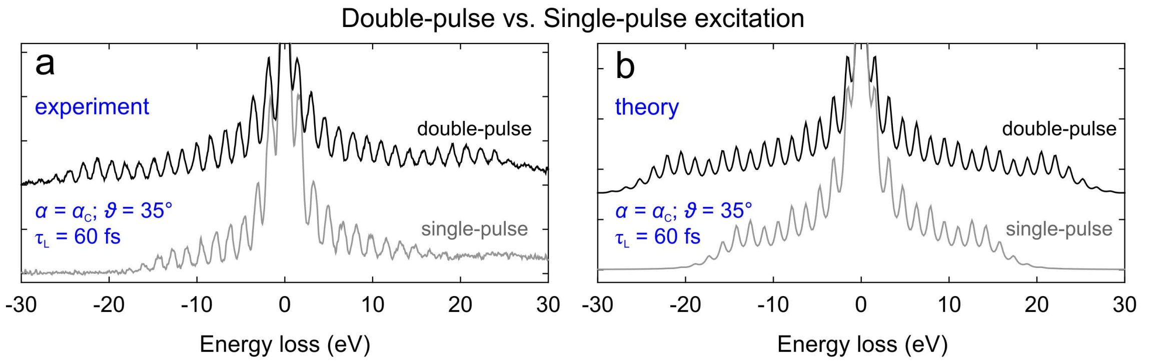

Supplementary Figure 9: Measured (panel a) and calculated (panel b) EEL spectra for illumination using a single-pulse (grey curves) or a double-pulse (black curves) configuration.

electronic states in the material (that is, electrons can originate from different initial states in the cathode, such resulting in a finite inhomogeneous distribution of electron energies). Thus, the photoelectrons reach the sample with slightly different velocities. The FWHM of the ZLP is thus mainly determined by the statistical distribution of the speed of single-electrons wavepackets when averaged over the acquisition time (several seconds) of the energy spectrum (for a repetition rate of 1 MHz this means millions of events). This can be seen in Supplementary Figure 10 below, where we have used a Voigt function to fit a representative measured ZLP spectrum. The best fit gives a Gaussian broadening of 1.16 eV and a Lorentzian broadening of about 10 meV, whereas constraining the Lorentzian FWHM to larger values results in a clearly poor fitting.

In this scenario, the inhomogeneous temporal spread of the measured electron pulse over the acquisition time would be therefore determined by the statistical distribution of the time of arrival at the sample. For electrons with energies distributed around 200 keV in the ± 0.5 eV range and over a source-to-sample distance of about 1 m, it amounts to about 216 fs. The total temporal duration of the electron pulse is then obtained by the geometric average of the inhomogeneous temporal spread and the homogeneous temporal duration of the electron wave function (the intrinsic longitudinal coherence).

To address the intrinsic degree of temporal coherence of the single-electron wave function in our setup, it is important to consider that the electron probe used in the microscope is obtained by photoemission from a photocathode following illumination with fs UV pulses. In the regime of having a single-electron wavepacket at each pulse, the energy bandwidth of the wave function of the photoemitted electrons would be determined by the bandwidth ΔE of the excitation UV pulses, which is on the order of tens of meV. Applying the uncertainty principle in the time-energy domain, we have $\sigma_E \sigma_t \geq \hbar/2$, where $\sigma_E = \Delta E/\sqrt{8\ln 2}$. Defining the coherence time ξ_t as twice the σ_t-value of the wave function (that is, $\xi_t = 2\sigma_t$, see Ref. 2), we obtain $\xi_t \geq \hbar/\sigma_E = \hbar\sqrt{8\ln 2}/\Delta E$, which turns out to be on the order of tens of femtoseconds.

To tentatively extract a rough estimate of the intrinsic longitudinal temporal coherence of the single-electron wave function, we used the experimental map in Fig. 4. Consider the envelope of the intensity profile of the high-energy spectral sidebands as a function of the delay time between the two pulses, $\Delta_2 - \Delta_1$. This envelope could be interpreted as the tail of a Gaussian function centered at $\Delta_2 - \Delta_1 = 0$, and it would represent the homogeneous broadening of the single-electron wavepacket. The least-square fit of the data for sidebands at $\ell = 13$, 14, and 15 with a Gaussian function results in values of the intrinsic longitudinal coherence that vary

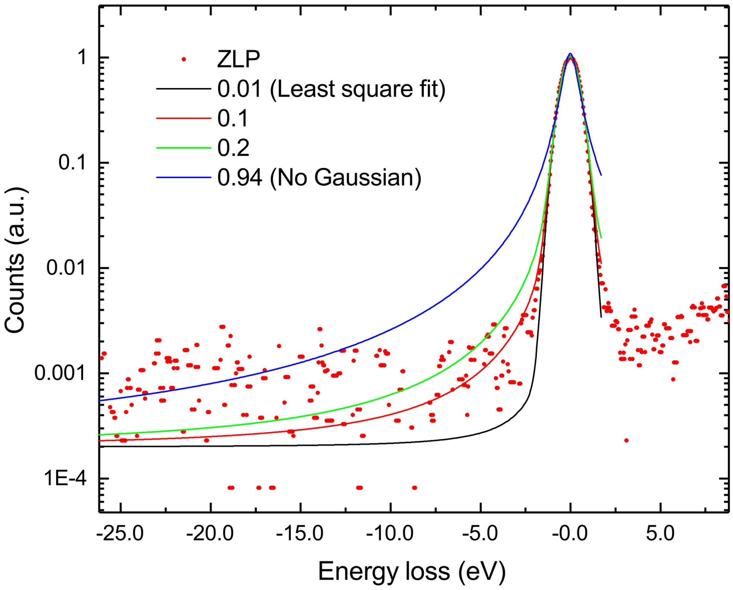

Supplementary Figure 10: Determination of Gaussian and Lorentzian broadening in a typically measured ZLP spectrum. The black curve is a best fit with a Voigt function, whereas the red and green curves represent the fit when the Lorentzian broadening is constrained to be 100 meV and 200 meV, respectively. The blue curve is the best fit using a purely Lorentzian lineshape.

between 52 and 78 fs, with an uncertainty of about ±10 fs. These values are in agreement with the estimate proposed above based on the uncertainty principle. In fact, considering an energy bandwidth $\Delta E = 20\,\mathrm{meV}$ of the wave function of the photoemitted electrons, as given by the bandwidth of the excitation pulses, the longitudinal temporal coherence would be $\xi_t = 77.5\,\mathrm{fs}$.

Considering that the free-space propagation from the source to the sample does not affect the longitudinal coherence, the single-electron wavepacket interacting with the light at the silver mirror maintain an intrinsic temporal coherence comparable with the temporal separation between the two optical pulses as used in the double-pulse scheme implemented here, which is the condition required for our experiment to work. Similar considerations apply when using two phase-locked fs x-ray pulses, further extending the validity of our coherent control scheme to the zeptosecond timescale.

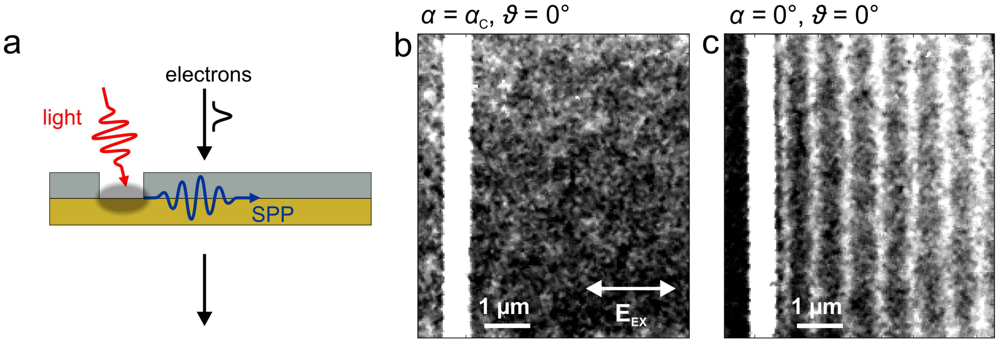

Supplementary Figure 11: Visualization of propagating surface-plasmon polaritons. (a) Schematic representation of the generation of surface plasmon polaritons by optical illumination at the edge of a nanocavity carved in the Ag layer. (b) Experimentally measured energy-filtered image for a sample orientation such to have a vanishing electron-light interaction ($\alpha = \alpha_C$ and $\vartheta = 0°$). (c) Experimentally measured energy-filtered image for a sample orientation such to have a non-negligible electron-light interaction ($\alpha = 0°$ and $\vartheta = 0°$). Only in the latter configuration a position-dependent interference of the propagating light and SPP fields as mediated by the electron beam occurs giving rise to a spatially oscillating field amplitude that can be imaged in real-space.

Supplementary Note 8: Visualization and coherent control of surface-plasmon polaritons

In the present work we have described energy-momentum exchange between an electron beam and a semi-infinite light field. In light-assisted electron microscopy experiments, this kind of interaction would generally coexist with electron scattering from photo-excited surface-plasmon polaritons (SPPs) originating from a nanostructured sample. The theory developed in our work provides a complete framework for quantitatively describing both of these effects, allowing us to fully and unambiguously interpret the experimental results. As an illustration of this point, we consider a configuration in which the phase-controlled combination of the two mentioned interactions (direct light reflection and coupling to SPP modes) can be used to perform photon-assisted electron microscopy of propagating plasmon waves, thus providing direct access into the field of these surface modes. A SPP wave can be generated by optical illumination at the edge of a nanocavity carved in the Ag layer (see Supplementary Figure 11a). For our Ag film, SPPs at the illumination frequency move at a speed of $\sim 1-2\times10^8$ m/s, thus propagating over a distance of $\sim 10-20\,\mu$m during the electron pulse duration of ~ 300 fs. When light-electron interaction is negligible (for example, for $\alpha=\alpha_C$ and $\vartheta=0°$), only SPPs couple to the electrons, and consequently, the time average of the SPP-electron interaction measured in our experiment results in a spatially homogeneous contrast in the energy-filtered real-space electron distribution (see Supplementary Figure 11b). In contrast, when the light-electron interaction is non-negligible (for example, for $\alpha=0°$ and $\vartheta=0°$), a position-dependent interference of the propagating light and SPP fields gives rise to a spatially oscillating field amplitude that can be now imaged in real-space, revealing characteristic fringes of period equal to the plasmon wavelength (see Supplementary Figure 11c).

As shown in Fig. 6a-d in the manuscript, coherent control of these plasmonic near-fields can be achieved on a attosecond-nanometer scale by using the developed two-pulse scheme. This has been demonstrated on a plasmonic Fabry-Perot (FP) resonator nano-fabricated in the Ag layer according to the design shown in Supplementary Figure 12a. Two sets of nano-antenna arrays, similar to those employed in Ref. 3, couple the incident optical field to SPPs propagating as plane waves in the direction perpendicular to the arrays. This configuration provided an emission tunable with the polarization of the incident light and a strongly enhanced plasmonic field generated within the cavity. When adopting an experimental geometry that cancels the interaction with the semi-infinite field ($\alpha=\alpha_C$ and $\vartheta=0°$), the fundamental plasmon mode of the FP cavity at $\lambda_{\rm SPP}/2\approx 350$ nm can be solely imaged, as shown in Fig. 6a in the main text. The coherent control experiment is performed as schematically depicted in Supplementary Figure 12b. The two 60 fs-long optical pulses were separated by more than 100 fs in order to minimize the optical interference between them on the sample, and their delay was varied in steps of 334 as while simultaneously acquiring energy-filtered images of the plasmonic interference pattern.

Supplementary Note 9: Calculation of the coupling strength between electrons and x-rays

In the main text we have discussed the possibility to adopt our approach for controlling electron-photon interaction using photons of different energies, and therefore potentially reaching timescales below the attosecond regime by using x-ray beams. In particular, we have described the possibility to use pulsed x-rays at 777 eV reflected by a Au/Co multilayer mirror. As an important preliminary step, the thickness of the Au and Co layers has to be designed in order to have a significant reflectivity at the $L_{2,3}$ absorption edge of Co for an angle of incidence around 45°, so that the interaction with electrons is maximized. In Supplementary Figure 13 we plot the interaction strength β as a function of photon energy and incidence angle ϑ. Using 30 layers of 1.6-nm-thick cobalt spaced by 1-nm-thick gold layers, we calculate a substantial increase in β under 777 eV at 45° light incidence.

As an important point regarding the feasibility of the experiment, we calculate here the absorbed X-ray energy per illuminated layer in the proposed structure. Considering that the multilayer has a reflection coefficient $R=0.35$ for a 777-eV X-ray beam, and that the absorption coefficient μ for gold and cobalt at this energy is around $1.3\times10^5\,{\rm cm}^{-1}$,[4] the absorbed energy per unit volume is: $f^V_{\rm abs}=f_0(1-R)\mu$, where $f_0=5\,{\rm J\,cm}^{-2}$ is the incident fluence. In a single layer of gold or cobalt with thickness d ($d=1$ nm for gold and $d=1.6$ nm for cobalt), the absorbed energy per unit area would then be $f^A_{\rm abs}=f_0(1-R)\mu d$, which becomes $42.2\,{\rm mJ\,cm}^{-2}$ for the gold layer and $67.5\,{\rm mJ\,cm}^{-2}$ for the cobalt layer. These numbers are smaller than the typical values of tenths of ${\rm J\,cm}^{-2}$ for damage threshold in metals.

---

* Authors contributed equally
† E-mail: javier.garciadeabajo@nanophotonics.es
‡ E-mail: fabrizio.carbone@epfl.ch

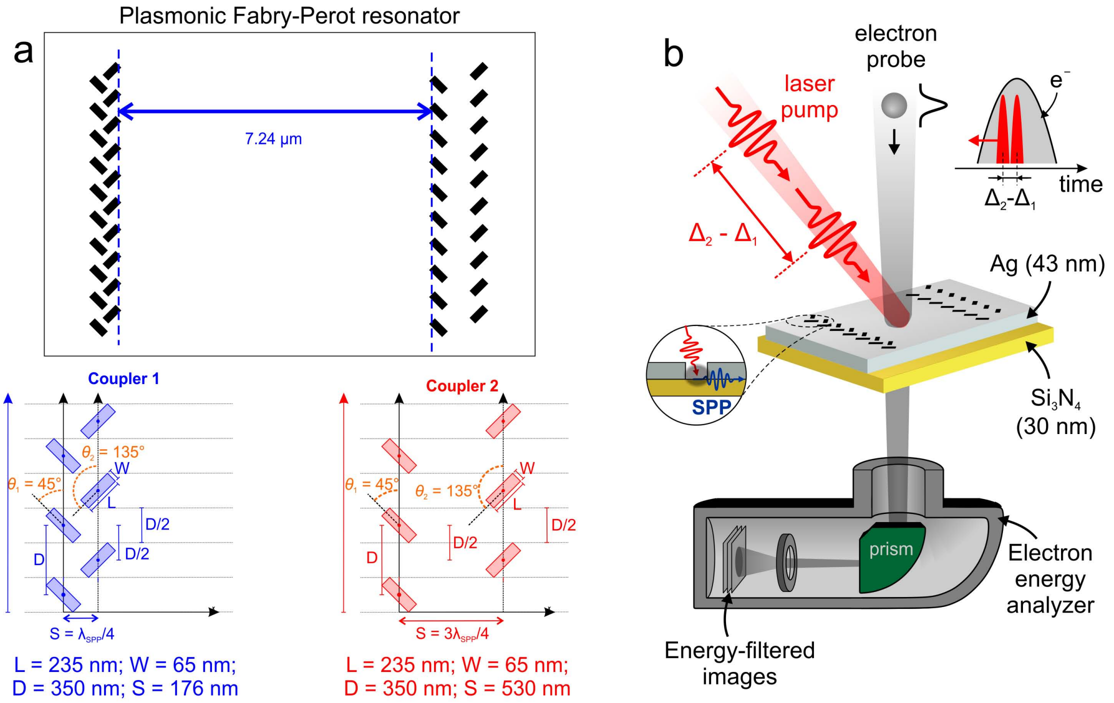

Supplementary Figure 12: Coherent control of plasmonic near-fields at attosecond-nanometer scale. (a) Design of a plasmonic Fabry-Perot (FP) resonator nano-fabricated in the Ag layer. (b) Schematic description of the two-pulse scheme for the coherent control of plasmonic fields.

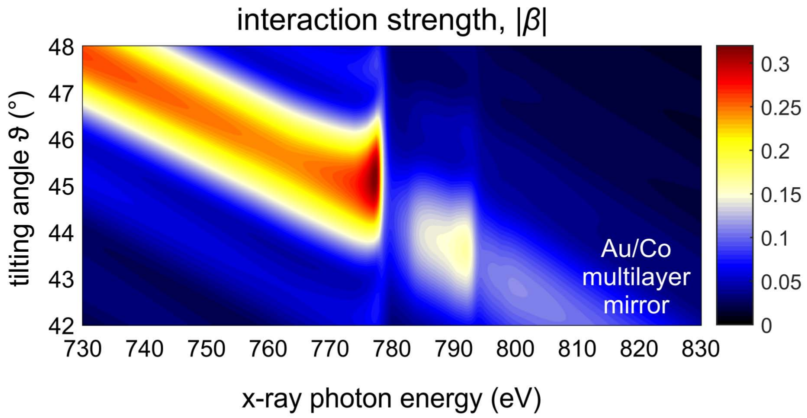

Supplementary Figure 13: Calculated interaction strength $|\beta|$ as a function of photon energy and incidence angle ϑ for a Au/Co multilayer mirror made of 30 layers of 1.6-nm-thick cobalt spaced by 1-nm-thick gold layers.

Supplementary References

[1] F. J. García de Abajo, Rev. Mod. Phys. **82**, 209-275 (2010).
[2] P. Baum, Chem. Phys. **423**, 55-61 (2013).
[3] J. Lin, J.P. Balthasar Mueller, Q. Wang, G. Yuan, N. Antoniou, X.-C Yuan, F. Capasso, Science **340**, 331-334 (2013).
[4] https://physics.nist.gov/PhysRefData/FFast/html/form.html,
